# Efficacy evaluation of acupuncture combined with Liujunzi Decoction in the treatment of functional dyspepsia: A protocol of randomized controlled trial

**DOI:** 10.1097/MD.0000000000024528

**Published:** 2021-02-26

**Authors:** Yulan Zhang, Lihong Bian, Huan Long, Weichen Zhang, Yuqiong Hu

**Affiliations:** a Fifth Hospital in Wuhan; b Wuhan Children's Welfare Home, Wuhan, Hubei Province, China.

**Keywords:** acupuncture, functional dyspepsia, Liujunzi Decoction, protocol, randomized controlled trial

## Abstract

**Background:**

Functional dyspepsia (FD) is a common and frequently-occurring disease in internal medicine. It is known that Liujunzi decoction and acupuncture are widely used in the treatment of FD, but there are few studies on the combination of Liujunzi decoction and acupuncture in the treatment of FD, and its safety and efficacy are still controversial. Therefore, the purpose of this study is to evaluate the efficacy and safety of acupuncture combined with Liujunzi decoction in the treatment of FD.

**Methods:**

We designed a prospective randomized controlled trial. The study protocol was approved by the Clinical Research Ethics Committee of our hospital. Patients with FD were randomly assigned to the treatment group of acupuncture combined with Liujunzi Decoction (the experimental group) and the treatment group of Liujunzi Decoction (the control group) in a ratio of 1:1. Outcome indicators were Nepean Dyspepsia Index, the MOS item short from health survey, and adverse reactions. Finally, SPSS 18.0 software would be used for statistical analysis of the data.

**Discussion:**

This study will evaluate the efficacy and safety of acupuncture combined with Liujunzi Decoction in the treatment of FD and provide clinical basis for the use of acupuncture combined with Liujunzi Decoction in the treatment of FD.

**OSF Registration number:**

DOI 10.17605/OSF.IO/67GKN

## 1 Introduction

Functional dyspepsia (FD), a common gastroduodenal disease without organic lesions, is mainly characterized by upper abdominal pain, belching, nausea, vomiting, loss of appetite, and other symptoms.^[1–3]^ An investigation shows that the prevalence rate of FD in the world is between 10% and 30%, and the incidence rate of FD in Asia is between 8% and 23%. The most common symptom of FD patients is postmeal distress syndrome, accounting for 62.8% of FD.^[4,5]^ FD can occur at all ages and is related to lifestyle, diet, mood, and other factors.^[6]^ Because FD is difficult to cure and has a high recurrence rate, it leads to the long-term existence of symptoms, which seriously affects the quality of life of patients, consumes a lot of medical resources, and brings a great burden to patients and the social medical system.^[7,8]^

At present, the clinical treatment of FD is mainly to protect gastric mucosa, inhibit acid, promote gastrointestinal motility, and other symptomatic treatments. For example, Proton pump inhibitors,^[9]^ Histamine 2 receptor antagonist,^[10]^ and gastric motility drugs^[11]^ can improve the symptoms of FD, but long-term use can easily lead to adverse reactions.^[12]^ In recent years, traditional Chinese medicine has shown its unique advantages in the treatment of FD and has achieved good results. Studies have found that Liujunzi Decoction can effectively improve anorexia in patients with lung cancer^[13]^ and has also achieved good efficacy in the treatment of FD.^[14]^ The clinical efficacy of acupuncture on FD has also been confirmed by a number of clinical trials, which can improve patients’ dyspepsia symptoms and is no less effective than conventional western medicine treatment.^[15,16]^ Currently, there is no clinical study on acupuncture combined with Liujunzi Decoction in the treatment of FD. Therefore, we designed this randomized controlled trial to evaluate the efficacy and safety of acupuncture combined with Liujunzi decoction in the treatment of FD.

## 2 Materials and methods

### 2.1 Study design

**Figure 1:**
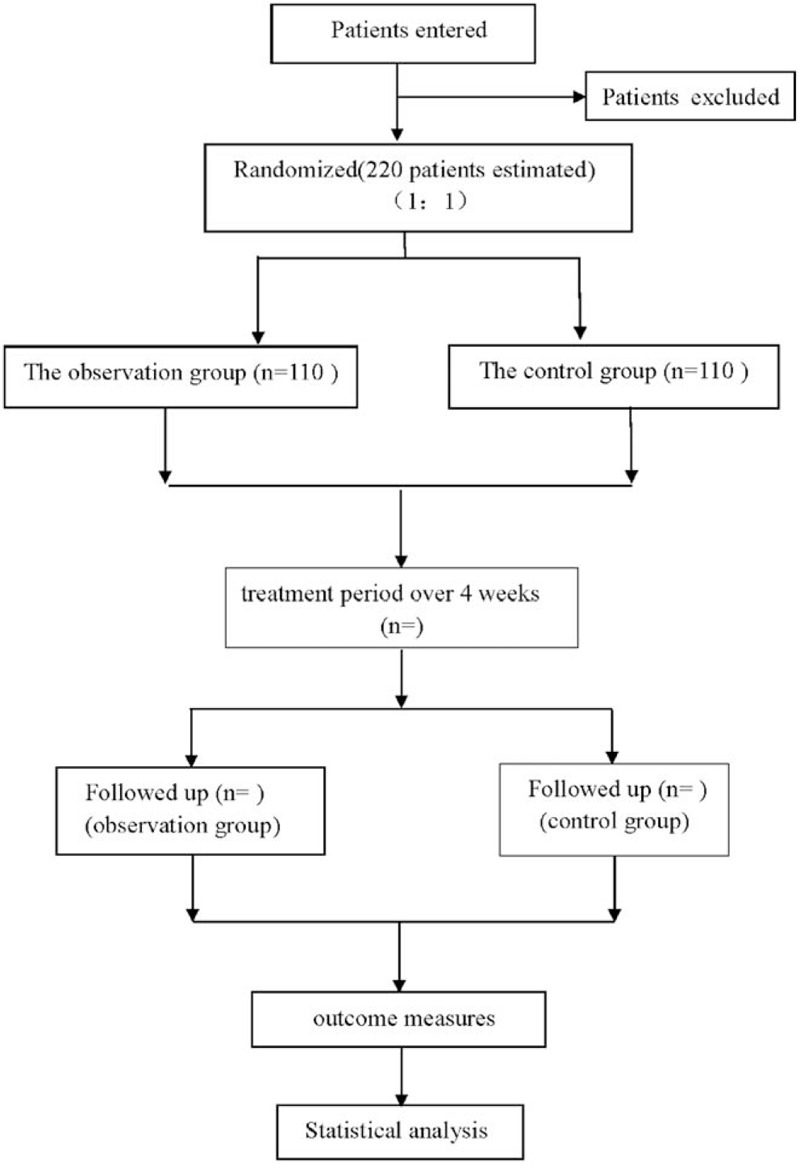
Flow diagram.

This is a single-center, prospective, randomized controlled trial protocol, which is to investigate the efficacy and safety of acupuncture combined with Liujunzi Decoction in the treatment of FD. This study protocol will follow the Acupuncture Clinical Trial Intervention Reporting Standards,^[17]^ and improve the study protocol according to the Consolidated Standards of Reporting Trials (CONSORT). The flow chart is shown in Figure 1.

### 2.2 Ethics and registration

This research scheme is in line with *the Helsinki Declaration* and has been approved by the Clinical Research Ethics Committee of our hospital. In addition, it has been registered on open science framework (Registration number: DOI 10.17605/OSF.IO/67GKN). We will fully inform patients and their families of the potential risks of this study. Patients and their families need to sign a written informed consent form to show that they understand the research program and are willing to participate in the trial. Patients are free to choose whether to continue the trial at any time during the study.

### 2.3 Sample size

In this study, the sample size was estimated according to the score of Nepean Dyspepsia Index (NDI) and the relevant literature was consulted. A study^[18]^ also adopted NDI as the outcome indicator, with the score of the experimental group being 90.028 ± 8.910 and the score of the control group being 86.040 ± 9.210. Set α = 0.05, unilateral test, β = 0.10, each group needs about 88 participants, the estimated withdrawal rate is 20%, each group will include 110 participants. The sample calculation formula is as follows:

$$n=n1=n2=\frac{{\left(u_{\alpha }+u_{\beta }\right)}^{2}\times \sigma^{2}}{\delta^{2}}\times 2$$

### 2.4 Patients

#### 2.4.1 Inclusion criteria

(1) Patients whose ages range from 18 to 65 years old;
(2) Patients who meet the diagnostic criteria for FD in Rome III;
(3) Patients whose gastroscopy showed normal results in past 1 year, and no other organic lesions were found in the gastrointestinal tract.
(4) Patients who did not take any stomach-stimulating drug for at least 15 days before the study, and did not participate in other clinical studies.

#### 2.4.2 Exclusion criteria

(1) Patients who have mental disorders or unconsciousness and are unable to express subjective discomfort symptoms, or patients with mental illness.
(2) Patients who suffer from malignant tumors, infectious diseases, and hemorrhagic diseases.
(3) Patients with severe primary diseases such as cardiovascular, liver, kidney, digestive tract, and hematopoietic system.
(4) Pregnant women, women who plan to be pregnant or breastfeeding.
(5) Patients with poor compliance.
(6) Patients who are unable to understand the research scheme or are unwilling to sign the informed consent form after explanation.

#### 2.4.3 Elimination and exfoliation standard

Those who meet one of the following conditions shall be included in the elimination cases:

1. Subjects who meet the included study but have not been tested;
2. Subjects who had mild adverse reactions during acupuncture treatment and were unwilling to continue treatment.

Those who meet one of the following conditions shall be included in exfoliation cases:

1. Patients with poor tolerance and poor compliance during acupuncture treatment, which will affect the evaluation of effectiveness and safety;
2. Patients who were withdrawn from the trial, lost to follow-up, or died due to other reasons before treatment was completed.

All cases of exfoliation are explained in the case report.

### 2.5 Recruitment strategy and distribution plan

We will recruit participants through a variety of approaches. Firstly, trained gastroenterologists will screen participants among the visiting patients. Secondly, post recruitment information on the hospital lobby or bulletin board and use the Internet and the media to publish recruitment information in newspapers, brochures, and WeChat platforms. For participants who meet the inclusion criteria, the purpose and program of this study shall be fully informed, and the informed consent signed by participants shall be obtained.

In this study, participants were randomly divided into the experimental group and the control group in a 1:1 ratio. Random numbers were generated by statisticians using a computer software program and placed in opaque envelopes that were handed to participants by study coordinators. In consideration of the practical nature of acupuncture, participants and operating physician may be aware of the randomization. But the evaluators of the findings and the statisticians who analyzed the data were unaware of the distribution.

### 2.6 Intervention measures

Patients in both groups were told what to do during the trial, including diet, exercise, etc.

Control group: Liujunzi decoction was provided by the pharmacy of the hospital, which mainly consisted of 10 g Dang Shen (Radix Codonopsis), 15 g Bai Zhu (Rhizoma Atractylodis Macrocephalae), 10 g Fu Ling (Poria), 10 g Ban Xia (Rhizoma Pinelliae), 10 g Chen Pi (Pericarpium Citri Reticulatae), and 6 g baked Gan Cao (Radix Glycyrrhizae). Liujunzi decoction was decocted in water for oral dose twice a day for 6 consecutive days, and there will be a 1–day interval after that. Patients should take medicine following the rules above for 4 weeks.

Experimental group: combined acupuncture treatment on the basis of the control group. The patient was placed in a comfortable position, and the trained Chinese medicine physician performed acupuncture on the fully exposed operative site (Zusanli (ST36), Neiguan (PC6), Zhongwan (CV12), Tianshu (ST25), Sanyinjiao (SP6), Diji (SP8), Yanglingquan (GB34), and Weishu (BL21) with the sterilized acupuncture needles (25–40 mm in length and 0.25 mm in diameter). The depth of acupuncture was 5 mm to 30 mm, and the patient had a sense of “arrival of Qi” (acid, distension, numbness, and other senses occurring in the acupuncture part). The needles were retained for 20 minutes each time. The treatment should be performed 3 times a week, lasting for 4 weeks.

After the end of the treatment course, participants were followed up by telephone twice a week for a total of 4 weeks, during which participants were instructed not to conduct other interventional such as drugs.

### 2.7 Observation Index

1. NDI^[19]^: NDI includes 2 parts: Nepean Dyspepsia Symptom Index and Nepean Dyspepsia Life Quality Index. The Nepean Dyspepsia Symptom Index score consisted of 15 items, and each item has 3 items, including attack frequency (0–4 points), extent (0–5 points), and influence degree (0–4 points). The higher the score is, the more severe the patient's symptoms are. The Nepean Dyspepsia Life Quality Index score consists of 25 items. First, the original score of each item is added up to S, then the minimum score in total is M (the sum of the minimum possible scores of each item), and the maximum score in total is R, with the total score = 100-[(s-m) /R]×100. The higher the score is, the higher the quality of life of the patient is. Efficacy index = (points before treatment - points after treatment)/points before treatment ×100%. Criteria for curative effect: ① Clinical recovery: the main symptoms and signs disappeared or basically disappeared, the curative effect index ≥95%; ② Significant effects: the main symptoms and signs were significantly improved, 70%≤ the curative effect index <95%; ③ Effectiveness: the main symptoms and signs were significantly improved, 30%≤ the curative effect index <70%; ④ Ineffectiveness: the main symptoms and signs were not significantly improved, or even aggravated, the curative effect index <30%.
2. The MOS item short from health survey (SF-36): this scale summarizes the quality of life of patients from eight aspects: physiological function, role-physical, bodily pain, general health, vitality, social functioning, role-emotional, mental health. The conversion formula of the scale score is [(actual score-lowest possible score)/general average possible score] × 100.
3. Adverse reactions: including intolerance during the test, dizziness, local redness and swelling caused by acupuncture, etc.

### 2.8 Data collection and management

The patients were followed up by 2 assistants by telephone for 4 weeks after treatment, and the data were collected and recorded. Personal information about potential participants and registered participants will be collected, shared, and stored in a separate storeroom to protect pre-test, in-test, and post-test confidentiality. People outside this research group do not have access to relevant data.

### 2.9 Statistical analysis

The collected data were statistically analyzed by SPSS 18.0 software. Chi-square test was used for counting data; the mean ± standard deviation ($\bar{x}\pm \text{S}$) was used for the measurement data, the independent sample *T*-test was used for the normal distribution, and the Mann-Whitney *U* test was used for the skewed distribution. The difference was considered statistically significant when *P* < .05.

## 3 Discussion

FDS include postprandial distress syndrome and epigastric pain syndrome, the former is characterized by early satiety and postprandial fullness, while the latter is characterized by upper abdominal pain and burning sensation.^[20]^ The pathogenesis of FD is not fully understood, but is currently known to be related to increased visceral sensitivity,^[21]^ abnormal gastric motility,^[22]^ brain-gut axis dysfunction,^[23]^ psychosocial factors,^[24]^ etc. There are many ways to treat FD, among which acupuncture is accepted by more and more patients due to its small side effects and good therapeutic effect.

Acupuncture is widely used in clinical practice, such as functional constipation,^[25]^ gastroesophageal reflux disease,^[26]^ and FD.^[27]^ Chen et al.^[28]^ found in animal experiments that electroacupuncture Zusanli can enhance gastrointestinal movement by improving vagus nerve activity. Wang Liu et al^[29]^ believed that the reason why electroacupuncture can regulate the gastric motility of FDD rats may be related to the change of NMDAR activity in central DMV (dorsal motor nucleus of the vagus) area of rats, resulting in the decrease of peripheral serum NO content. According to Wang et al,^[30]^ acupuncture can improve the symptoms of FD patients, which is related to the inactivation of brainstem, insula, thalamus, and hypothalamus. Zhou et al^[31]^ found through systematic analysis that acupuncture can significantly improve the symptoms of FD patients and improve their quality of life. Liujunzi decoction is a classic ancient prescription of traditional Chinese medicine, in which Dang Shen (Radix Codonopsis) invigorating spleen and replenishing Qi, Bai Zhu (Rhizoma Atractylodis Macrocephalae) and Fu Ling (Poria) invigorating spleen for eliminating dampness, Chen Pi (Pericarpium Citri Reticulatae) and Ban Xia (Rhizoma Pinelliae) drying dampness, resolving phlegm and regulating the flow of Qi and the middle Jiao, baked Gan Cao (Radix Glycyrrhizae) harmonizing the property of all herbs. All herbs should be used together, removing both its roots cause and symptoms, giving full play to the effects of strengthening the spleen and stomach and regulating the flow of Qi to alleviate pain. Zhao et al^[32]^ found that Enterochromaffin 5HT3r (cell-5-hydroxytryptamine 3 receptor) signal transduction was inhibited and visceral hypersensitivity was reduced in patients treated with Liujunzi decoction. Zhang et al^[33]^ showed that Liujunzi decoction could improve the symptoms of FD patients with spleen-deficiency and qi-stagnation syndrome. Some studies have confirmed that the curative effect of acupuncture combined with Liujunzi decoction in the treatment of FD is better than that of Liujunzi decoction alone.^[34]^ However, due to the lack of related research, there is no final conclusion on the combination of the two. Therefore, this study evaluates the efficacy and safety of acupuncture combined with Liujunzi decoction in the treatment of FD, in order to provide a basis for the promotion of this treatment in clinic.

This study is a single-center randomized controlled study, and the population included is regional, which may affect the results of the study. Secondly, due to the treatment of acupuncture, it is impossible to achieve strict double blindness, and the results of the study may be affected. In addition, the follow-up time of this study is short, so it is impossible to know the lasting effect of the treatment regimen. Therefore, more large sample, multicenter, and long-term follow-up studies are needed to confirm the therapeutic effect of acupuncture combined with Liujunzi decoction.

## Author contributions

**Data curation:** Yulan Zhang, Lihong Bian.

**Funding acquisition:** Huan Long.

**Investigation:** Yulan Zhang, Lihong Bian.

**Literature retrieval:** Yulan Zhang and Lihong Bian.

**Recruiting patients:** Yulan Zhang and Lihong Bian.

**Software:** Weichen Zhang, Yuqiong Hu.

**Supervision:** Huan Long.

**Writing – original draft:** Yulan Zhang, Lihong Bian.

**Writing – review & editing:** Yulan Zhang, Huan Long.

## References

[1] Enck P Azpiroz F Boeckxstaens G. Functional dyspepsia. Nat Rev Dis Primers 2017;3:17081. 29099093 10.1038/nrdp.2017.81

[2] Madisch A Andresen V Enck P. The diagnosis and treatment of functional dyspepsia. Dtsch Arztebl Int 2018;115:222–32. 29669681 10.3238/arztebl.2018.0222 PMC5938438

[3] Ford AC Mahadeva S Carbone MF. Functional dyspepsia. Lancet 2020;396:1689–702. 33049222 10.1016/S0140-6736(20)30469-4

[4] Barberio B Mahadeva S Black CJ. Systematic review with meta-analysis: global prevalence of uninvestigated dyspepsia according to the Rome criteria. Aliment Pharmacol Ther 2020;52:762–73. 32852839 10.1111/apt.16006

[5] Ghoshal UC Singh R Chang FY. Epidemiology of uninvestigated and functional dyspepsia in Asia: facts and fiction. J Neurogastroenterol Motil 2011;17:235–44. 21860815 10.5056/jnm.2011.17.3.235 PMC3155059

[6] Zhang SS Zhao LQ. Expert consensus on the diagnosis and treatment of functional dyspepsia (2017). China J Tradit Chin Med Pharm 2017;32:2595–8.

[7] Lacy BE Weiser KT Kennedy AT. Functional dyspepsia: the economic impact to patients. Aliment Pharmacol Ther 2013;38:170–7. 23725230 10.1111/apt.12355

[8] Moayyedi P Mason J. Clinical and economic consequences of dyspepsia in the community. Gut 2002;50:10–2. 10.1136/gut.50.suppl_4.iv10 PMC1867687 11953338

[9] Pinto-Sanchez MI Yuan Y Bercik P. Proton pump inhibitors for functional dyspepsia. Cochrane Database Syst Rev 2017;3:CD011194. 28271513 10.1002/14651858.CD011194.pub2 PMC6464600

[10] Seno H Nakase H Chiba T. Usefulness of famotidine in functional dyspepsia patient treatment: comparison among prokinetic, acid suppression and antianxiety therapies. Aliment Pharmacol Ther 2005;21:32–6. 15943844 10.1111/j.1365-2036.2005.02471.x

[11] Yang YJ Bang CS Baik GH. Prokinetics for the treatment of functional dyspepsia: Bayesian network meta-analysis. BMC Gastroenterol 2017;17:83. 28651565 10.1186/s12876-017-0639-0 PMC5485548

[12] Fossmark R Martinsen TC Waldum HL. Adverse effects of proton pump inhibitors-evidence and plausibility. Int J Mol Sci 2019;20:5203. 10.3390/ijms20205203 PMC6829383 31640115

[13] Sun LL Lai HZ Chen ZZ. Modified liujunzi decoction alleviates chemotherapy-induced anorexia in advanced non-small cell lung cancer: a propensity score matched case-control study. Chin J Integr Med 2020;26:256–62. 31970675 10.1007/s11655-020-3185-5

[14] Li CH. Clinical observation on treating functional dyspepsia 4with the Liujunzi decoction. Clin J Chin Med 2015;7:120–1.

[15] Jin Y Zhao Q Zhou K. Acupuncture for functional dyspepsia: a single blinded, randomized, controlled trial. Evid Based Complement Alternat Med 2015;2015:904926. 26294930 10.1155/2015/904926 PMC4534622

[16] Zhang J Liu Y Huang X. Efficacy comparison of different acupuncture treatments for functional dyspepsia: a systematic review with network meta-analysis. Evid Based Complement Alternat Med 2020;2020:3872919. 32256643 10.1155/2020/3872919 PMC7106911

[17] MacPherson H Altman DG Hammerschlag R. Revised Standards for Reporting Interventions in Clinical Trials of Acupuncture (STRICTA): extending the CONSORT statement. J Evid Based Med 2010;3:140–55. 21349059 10.1111/j.1756-5391.2010.01086.x

[18] Zeng F Qin W Ma T. Influence of acupuncture treatment on cerebral activity in functional dyspepsia patients and its relationship with efficacy. Am J Gastroenterol 2012;107:1236–47. 22641307 10.1038/ajg.2012.53

[19] Jones MP Sato YA Talley NJ. The Nepean Dyspepsia Index is a valid instrument for measuring quality-of-life in functional dyspepsia. Eur J Gastroenterol Hepatol 2019;31:329–33. 30461521 10.1097/MEG.0000000000001314

[20] Futagami S Yamawaki H Agawa S. New classification Rome IV functional dyspepsia and subtypes. Transl Gastroenterol Hepatol 2018;3:70. 30363705 10.21037/tgh.2018.09.12 PMC6182037

[21] Kim BJ Kuo B. Gastroparesis and functional dyspepsia: a blurring distinction of pathophysiology and treatment. J Neurogastroenterol Motil 2019;25:27–35. 30509017 10.5056/jnm18162 PMC6326193

[22] Takahashi T. Interdigestive migrating motor complex -its mechanism and clinical importance. J Smooth Muscle Res 2013;49:99–111. 24662475 10.1540/jsmr.49.99 PMC5137267

[23] Browning KN Travagli RA. Central nervous system control of gastrointestinal motility and secretion and modulation of gastrointestinal functions. Compr Physiol 2014;4:1339–68. 25428846 10.1002/cphy.c130055 PMC4858318

[24] Geeraerts B Vandenberghe J Van Oudenhove L. Influence of experimentally induced anxiety on gastric sensorimotor function in humans. Gastroenterology 2005;129:1437–44. 16285945 10.1053/j.gastro.2005.08.020

[25] Zhang N Huang Z Xu F. Transcutaneous neuromodulation at posterior tibial nerve and ST36 for chronic constipation. Evid Based Complement Alternat Med 2014;2014:560802. 25431612 10.1155/2014/560802 PMC4238235

[26] Liu Z Lu D Guo J. Elevation of lower esophageal sphincter pressure with acute transcutaneous electrical acustimulation synchronized with inspiration. Neuromodulation 2019;22:586–92. 31136053 10.1111/ner.12967

[27] Xu F Tan Y Huang Z. Ameliorating effect of transcutaneous electroacupuncture on impaired gastric accommodation in patients with postprandial distress syndrome-predominant functional dyspepsia: a pilot study. Evid Based Complement Alternat Med 2015;2015:168252. 26064155 10.1155/2015/168252 PMC4433673

[28] Chen J Song GQ Yin J. Electroacupuncture improves impaired gastric motility and slow waves induced by rectal distension in dogs. Am J Physiol Gastrointest Liver Physiol 2008;295:G614–20. 18653722 10.1152/ajpgi.90322.2008

[29] Wang L Shen GM Wang H. The effects of electroacupuncture at shu and mu points of stomach on gastric motility, the NMDA of vagus nerve dorsal nucleus and serum NO expression in functional dyspepsia rats. Chin Acupunct Moxib 2018;38:285–90. 10.13703/j.0255-2930.2018.03.016 29701047

[30] Wang YP Herndon CC Lu CL. Non-pharmacological approach in the management of functional dyspepsia. J Neurogastroenterol Motil 2020;26:6–15. 31751504 10.5056/jnm19005 PMC6955193

[31] Zhou W Su J Zhang H. Efficacy and safety of acupuncture for the treatment of functional dyspepsia: meta-analysis. J Altern Complement Med 2016;22:380–9. 27028618 10.1089/acm.2014.0400

[32] Zhao J Zhao L Zhang S. Modified Liu-Jun-Zi decoction alleviates visceral hypersensitivity in functional dyspepsia by regulating EC cell-5HT3r signaling in duodenum. Ethnopharmacol 2020;250:112468. 10.1016/j.jep.2019.112468 31836517

[33] Zhang S Zhao L Wang H. Efficacy of modified Liujunzi Decoction on functional dyspepsia of spleen-deficiency and qi-stagnation syndrome: a randomized controlled trial. BMC Complement Altern Med 2013;13:54. 23453018 10.1186/1472-6882-13-54 PMC3599864

[34] Zhu RF Luo Y. Efficacy of acupuncture combined with liujunzi decoction in treatment of functional dyspepsia with spleen deficiency and qi stagnation. J Anhui Univ Chin Med 2016;35:51–3.

